# Supplementary material for: PLOS Computational Biology 2014 Reviewer Thank You
Source: PLoS Comput Biol. 2015 Feb 27;11(2):e1004184. doi: 10.1371/journal.pcbi.1004184 (PMC4344216; doi:10.1371/journal.pcbi.1004184)
Supplement: S1 Reviewer List — (PDF) [file pcbi.1004184.s001.pdf]

PLOS | COMPUTATIONAL BIOLOGY

PLOS Computational Biology would like to thank all those who reviewed on behalf of the journal in 2014:

Scott Aaronson
Larry Abbott
Pia Abel zur Wiesch
Maria Abou Chakra
Laith Abu-Raddad
Luigi Acerbi
Pablo Achard
Achilleas Achilleos
Jörg Ackermann
Daniel Acuna
Christoph Adami
Boris Adryan
Stein Aerts
Pankaj Agarwal
Amit Aggarwal
Matthew Agler
Smita Agrawal
Cendra Agulhon
Amir Aharoni
Yashar Ahmadian
Alaa Ahmed
Yong-Yeol Ahn
Sunil Ahuja
Christopher Aiken
Elizabeth Ailes
Antti Airola
Edoardo Airoldi
Laurence Aitchison
Thomas Akam
Joshua Akey
Eyal Akiva
Sahar Akram
Athena Akrami
Aleksei Aksimentiev
Tomas Alarcon
Larissa Albantakis
John Albeck
Réka Albert
Martí Aldea
Bree Aldridge
Max Alekseyev
Helen Alexander
Boian Alexandrov
Emil Alexov
Samuel Alizon
Yannick Allanore
Patrick Aloy
Jeff Alstott
Grégoire Altan-Bonnet
Christian Althaus
Russ Altman
Philipp Altrock
Mario Alvarez
Rommie Amaro
Roee Amit
Greg Amoutzias
Eva Amsen
Gary An
Sophia Ananiadou
John Anderson
Bart Anderson
Alexander Anderson
Paul Anderson
Marti Anderson
Ole Andreassen
R. Andrew
Steven Andrews
Ioan Andricioaei
Rustom Antia
Shunsuke Aoki
Kazuhiro Aoki
Juan Aparicio
Udayan Apte
Johan Åqvist
Salva Ardid
Fernando Arenzana-Seisdedos
Jacob Arguello
Nimalan Arinaminpathy
Stephane Aris-Brosou
Anton Arkhipov
Roger Armen
Alain Arneodo
Manimozhiyan Arumugam
Vinayagam Arunachalam
Lars Arvestad
Hugues Aschard
Giorgio Ascoli
Roman Ashauer
Gregory Ashby
Michael Assaf
Anand Asthagiri
Lauren Atlas
Benjamin Audit
Yurii Aulchenko
Kari Auranen
Erik Aurell
Bruno Averbeck
Ahmet Ay
Nihat Ay
Sandro Azaele
Rony Azouz
Eugenio Azpeitia

Madan Babu
Nicolas Bacaer
Brian Bachmann
Roland Baddeley
Gary Bader
Steven Baer
Vineet Bafna
Ivet Bahar
Wadie Bahou
Bahador Bahrami
Jane Bai
Lu Bai
Timothy Bailey
Chris Bailey-Kellogg
James Baish
Chris Bakal
Matthew Baker
Brian Baker
Ruth Baker
Chris Baker
Gabor Balazsi
Vincent Balter
Nilesh Banavali
Leah Band
Sourav Bandyopadhyay
Julio Banga
Edward Banigan
Claudia Bank
Shweta Bansal
Mukul Bansal
Svetlana Baoukina
Omri Barak
Yael Baran
Virginia Barbour
Alessandro Barducci
Lee Bardwell
Stephen Barigye
Debashis Barik
Ziv Bar-Joseph
Ernest Barreto
Adam Barrett
Xavier Barril
Simon Barthelme
Ed Bartlett
Frederic Bartumeus
Anastasia Baryshnikova
Patricia Bassereau
Philippe Bastiaens
Ugo Bastolla
Alex Bateman
Paul Bates
Mark Bathe
Francesco Battaglia
Alexis Battle
J. Fernando Bazan
Jason Bazil
Georgii Bazykin
Martin Beck
Jeff Beck
Oliver Beckstein
Attila Becskei
Michael Beer
Randy Beer
Niko Beerenwinkel
John Beggs
Chase Beisel
Alexander Beliaev
Pascal Belin
George Bell
Nicola Bellomo
Joost Beltman
Pedro Beltrao
Jan Benda
Robert Bender
Philip Benfey
Craig Benham
Asa Ben-Hur
Yoav Benjamini
Matthew Bennett
Richard Benninger
Sliman Bensmaia
Katie Bentley
Philipp Berens
Igor Berezovsky
Howard Berg
Bonnie Berger
Joshua Berke
Ari Berkowitz
Samuel Bernard
Marti Bernardo-Faura
Monica Berrondo
Anna Bershteyn
Alex Best
Robert Best
Matthias Bethge
Andreas Beyer
Pratik Bhagunde
Upinder Bhalla
Vikas Bhandawat
Nita Bharti
Sudin Bhattacharya
Tanmoy Bhattacharya
Joseph P. Bielawksi
Elaine Bignell
Ewan Birney
Inanc Birol
David Biron
Marc Birtwistle
Prakash Bisen
Jennifer Bizley

Nils Blüthgen
Jeffrey Blanchard
Romain Blanc-Mathieu
Francois Blanquart
Leonidas Bleris
Michael Blinov
Spencer Bliven
Jesse Bloom
Rainer Boeckmann
Joschka Boedecker
Lies Boelen
Pierre-Yves Boelle
Rafal Bogacz
Markus Böl
Peter Bond
Josh Bongard
Maciej Boni
Richard Bonneau
Roger Bonnecaze
Valerie Bonnelle
Susan Bonner-Weir
Erie Boorman
Martin Bootsma
Alla Borisyuk
Peer Bork
Alexander Borst
Giovanni Bosco
Peter Bossaerts
David Botstein
Maurizio Botta
Arezki Boudaoud
Eric Bouhassira
Anne-Laure Boulesteix
Robert Bourret
Bastien Boussau
Benjamin Bouvier
Paola Bovolenta
Grant Bowman
Gregory Bowman
Clive Bowsher
Philip Bradley
Tiago Branco
Aaron Brandes
Eduard Brandstätter
Hans Braun
A Braunstein
Johanni Brea
Michael Breakspear
Romulus Breban
Lindsay Bremner
Wieland Brendel
Eli Brenner
Michael Brenner
Naama Brenner
Steven Brenner
Paul Bressloff
Romain Brette
Ryan Brinkman
Russell Brinkworth
Abra Brisbin
James Briscoe
R. Broadhurst
Doriano Brogioli
Yana Bromberg
Ethan Bromberg-Martin
Mark Broom
Christine Brostjan
Emery Brown
Celeste Brown
Steven Brown
Kristin Brown
Sam Brown
C. Titus Brown
Chris Brown
Zachary Brown
Michael Browning
Ian Bruce
Frank Bruggeman
Søren Brunak
Nicolas Brunel
Aurelio Bruno
Jennifer Bryan
Mark Brynildsen
Gil Bub
Javier Buceta
Nicolae-Viorel Buchete
Gavin Buckingham
Christopher Buckley
Aidan Budd
David Budescu
Floris Buelens
Joerg Buescher
Lars Buesing
Michael Buhnerkempe
Alfonso Buil
Ralf Bundschuh
Anthony Burgard
Reinhard Bürger
Donald H. Burke
Anthony Burkitt
John Burnett
Jerry Busemeyer
Robin Bush
Harmen Bussemaker
Robert Butera
Daniel Butts
Markus Butz
Kevin Byrne
Kerstin Bystricky
Joana Cabral

Amedeo Caflisch
Mehmet Caglar
Gerard Cagney
Ronald Calabrese
Vince Calhoun
Curt Callan
Laurence Calzone
Carlos Camacho
Valentina Cambiano
Colin Campbell
Jeffrey Campion
Carmen Canavier
Jianhua Cang
Robert Cannon
Xiaoyi Cao
Lulu Cao
Emidio Capriotti
Ring T. Carde
Vincent Carey
Paolo Carloni
Heather Carlson
Ross Carlson
Jens Carlsson
Anders Carlsson
Carlos Carmona-Fontaine
Maura Casadio
Rita Casadio
Donatello Castellana
Brian Castle
Jordi Catalan
Simon Cauchemez
Gillian Caughey
James Cavanagh
Rachel Cavill
Jean-Baptiste Cazier
Michele Ceccarelli
Matteo Ceccarelli
Antonio Celani
Pablo Chacon
Maurice Chacron
Monica Chagoyen
Arup Chakraborty
Farida Chamchod
Hue Chan
Nagasuma Chandra
Chandramouli Chandrasekaran
Chia-en Chang
Jeffrey Chang
Luke Chang
Dennis Chao
Claudine Chaouiya
Michael Chappell
John Chappell
Catherine Charneski
Pep Charusanti
Steve Chase
Ishanu Chattopadhyay
Eleftheria Chatzimichail
Anuradha Chauhan
Jerome Chave
Mario Chavez
Tomas E Cheatham
Gal Chechik
Rong Chen
Alan Chen
Chia-Yen Chen
Xing Chen
Wei-Hua Chen
Han Chen
Junjie Chen
Jake Chen
I-Jen Chen
Quan Chen
Liang Chen
Wen Chen
Z Chen
Chao Cheng
Cheng Cheng
Jianlin Cheng
Elissa Chesler
Lily Cheung
Sung Wook Chi
Dante Chialvo
Vikram Chib
Lauren Childs
Francis Chin
Christophe Chipot
Julien Chiquet
Haridas Chirakkal
Nakul Chitnis
Lars Chittka
Sam Cho
Samuel Cho
Adrien Chopin
Ching-Shan Chou
Tom Chou
Gerardo Chowell
Beat Christen
David Christini
Carlton Chu
Charlie Chubb
Rumi Chunara
Ho-Ryun Chung
Gennady Churakov
Luca Ciandrini
Massimo Ciccozzi
Radoslaw Cichy
Marek Cieplak
Giovanni Ciriello
Patrick Cirino

Paul Cisek
Gerardo Cisneros
Stanca Ciupe
Jean Clairambault
Hannah Clapham
Tim Clark
Wyatt Clark
Connor Clark
Nicolas Clauvelin
Richard Clayton
Cecilia Clementi
John Clithero
Nicole Cloonan
Peter Clote
Christina Cobbold
Sarah Cobey
Simona Cocco
Luis Pedro Coelho
Ruben Coen-Cagli
Carlo Colantuoni
Claire Colas
James Cole
Michael Cole
Julio Collado-Vides
Frank Collart
Anne Collins
Giorgio Colombo
Ian Colrain
Lucy Colwell
Luca Comai
Andrew Connolly
Mark Cookson
Daniel Coombs
Jacob Cooper
Richard Cooper
Stephen Cooper
Anita Corbett
Russell Corbett-Detig
Victor Corces
Richard Cordaux
Courtney Corley
Bernat Corominas-Murtra
Manuel Corpas
Jose Correa-Basurto
Sonia Cortassa
Ivan Costa
Rui Costa
Frank Costantini
James Costello
Gary Cottrell
Zoe Cournia
Arthur Covert III
Lindsay Cowell
Susan Cox
Edward Cox
David Craig
Edmund Crampin
Rebecca Craven
Ross Cressman
Vittorio Cristini
Anton Crombach
Nicolas Crossley
Nicholas Croucher
Péter Csermely
José Cuesta
Qiang Cui
Juan Cui
Kathleen Cullen
Hermann Cuntz
Christine Currie
Christina Curtis
Carina Curto
Gennady Cymbalyuk
Tamás Czárán
Andras Czirok
Jack da Silva
Andreas Daffertshofer
Tal Dagan
Matteo Dal Peraro
Trevor Dale
Mark Daley
Yang Dan
Antoine Danchin
Thomas Dandekar
Astrid Dannenberg
Jayajit Das
Ranjan Dash
Torsten Dau
Stephen David
Maria Davidich
Diana David-Rus
Nathaniel Daw
Adriana Dawes
Troy Day
Peter Dayan
Marcus de Aguiar
Bouke de Boer
Alexandre de Brevern
Elke De Bruyne
Gianni De Fabritiis
Albert de Graaf
Wim de Grip
Bert de Groot
Willem de Haan
Monique de Jager
Jason de Koning
Gustavo de la Riva
Francisco De La Vega
Maurizio De Pittà
Jeroen de Ridder

Jaap de Roode
Bert De Rybel
Alfonso De Simone
Ive De Smet
Marco De Vivo
Willem de Vos
Anita de Waard
Charlotte Deane
Greg DeAngelis
Dominique Debanne
Gustavo Deco
Paolo Decuzzi
Peter Dedecker
Eric Deeds
Andrea Degaetano
Victor DeGruttola
Thomas Deisboeck
Annick Dejaegere
Domitilla Del Vecchio
Matthew DeLisa
Marcel den Nijs
Vincent Denef
Sophie Deneve
Christopher Denton
Bart Deplancke
Dori Derdikman
Hartmut Derendorf
Imre Derényi
Philippe Derreumaux
Christophe Dessimoz
Nicolas Destainville
Alain Destexhe
Marie Devaine
Michael DeWeese
Colin Dewey
Diego Di Bernardo
Bendetto di Martino
Aaron Diaz
Achim Dickmanns
Casey Diekman
Paul Dijkstra
Ruxandra Dima
Dobromir Dimitrov
David Dingli
Francisco Dionisio
Dennis Discher
Oliver Distler
Markus Dittrich
Mark Ditzler
Anna Divoli
Purushottam Dixit
Narendra Dixit
Hana Dobrovolny
Andy Dobson
Andrew Doig
Brent Doiron
Nikolay Dokholyan
Socrates Dokos
Eytan Domany
Wodarz Dominik
Stefano Donadio
Jiajia Dong
Christl Donnelly
Christian Donner
I. Dorigatti
Pemra Doruker
Zsuzsanna Dosztányi
Ivan Dotu
Stéphane Douady
Daniel Dougherty
John Doyle
Finn Drablos
Jeremy Draghi
John Drake
Brian Drawert
Jacqueline Dresch
Ron Dror
Shaul Druckmann
Jan Drugowitsch
Carsten Duch
Charles Duffy
Jacques Dumais
Geneviève Dupont
Anindya Dutta
Sandrine Duverne
Rhonda Dzakpasu
Jeffrey Eaton
David Eccles
Alexander Ecker
Miguel Eckstein
Michael Economo
Evan Economo
Leah Edelstein-Keshet
Robert Edgar
Paul Edlefsen
Andrew Edwards
Matthew Edwards
Felix Effenberger
Sol Efroni
Robert Egger
Charles Eggleton
Brian Egleston
Patrick Eichenberger
Michael Eickenberg
Thorsten Eierhoff
Robyn Eijlander
Roland Eils
Miriam Eisenstein
David Elad
Sami El-Boustani

Adrian Elcock
Wael El-Deredy
Marjet Elemans
Olivier Elemento
Mounya Elhilali
Arne Elofsson
Chris Elsik
Timothy Elston
Frank Emmert-Streib
Thierry Emonet
Heiko Enderling
Robert Endres
Jacob Engelmann
Sinead English
Bernhard Englitz
Daniel Ennis
Joshua Epstein
Harold Erickson
Bard Ermentrout
Marc Ernst
Jason Ernst
Eyal Ert
Sean Escola
Carlos Espinosa-Soto
Rino Esposito
Sylvie Estrela
Hari Eswaran
Ralf Everaers
Sebastian Eves-van den Akker
Eran Eyal
Eduardo Eyras
Elisa Fadda
James Faeder
François Fages
Adrienne Fairhall
Francesco Falciani
Martin Falcke
Daniel Falush
Hai Fang
Gang Fang
Christopher Fang-Yen
Christian Fankhauser
Hossein Farahani
Etienne Farcot
Dario Farina
Karoline Faust
Rachel Fearns
Michael Feig
Adam Feist
Jacob Feldman
Marcus Feldman
David Fell
Jérôme Feret
Neil Ferguson
Chrisantha Fernando
Evandro Ferrada
Matthew Ferrari
Dirk Fey
Ralf Ficner
Oliver Fiehn
Slawomir Filipek
Marta Filizola
James Finley
Christopher Fiorillo
Giacomo Fiorin
Daniel Fisher
Jasmin Fisher
David Fisman
Stephen Fleming
Daniel Fletcher
Alexander Fletcher
Nicolas Flores
Federico Fogolari
Brian Foley
Ariele Follis
M. Forest
Daniel Forger
Stefano Forli
Alex Fornito
Lucy Forrest
Kristoffer Forslund
Daryl Fougnie
Nicholas Foulkes
Jean-Baptiste Fournier
Douglas Fowler
Charless Fowlkes
Joanne Fox
Ingo Fründ
David Frakes
Christian Franck
Paul Francois
Marcos Frank
Steven Frank
Adam Frankish
Kevin Franks
Erik Fransen
Thomas Franz
Franca Fraternali
Tom Freeman
Jonathan Freund
Joel Freundlich
Erwin Frey
Saskia Freytag
Christian Frezza
Leonid Fridlyand
Hermann Frieboes
Caroline Friedel
Marc Friedlander
Aaron Friedman
Nir Friedman

Tobias Friedrich
Dmitrij Frishman
Karl Friston
Robert Froemke
Simon Frost
Gang Fu
Masami Fujiwara
Terrence Furey
Eileen Furlong
Nicholas Furnham
Robert Gütig
Terry Gaasterland
Fabrizio Gabbiani
Bruno Gaeta
Julien Gagneur
Roberto Galán
Patrick Gallagher
Jack Gallant
Jörg Galle
Alvaro Galli
Alberto Gandolfi
Sylvain Gandon
Olivier Gandrillon
Elad Ganmor
Victor Gannon
Vitaly Ganusov
Feng Gao
Fei Gao
Angel Garcia
Victor Garcia
Lana Garmire
Ruben Garrido Oter
Andrew Gartland
Paolo Gasti
Michael Gastner
M Carolyn Gates
James Gauld
Katharina Gaus
Sergey Gavrilets
Hao Ge
Nils Gehlenborg
Erol Gelenbe
Mikhail Gelfand
Guy Genin
Guy Georges
Mark Georgeson
Claude Gérard
Felipe Gerhard
Arne Gericke
Ulrich Gerland
Philip Gerlee
Samuel Gershman
Mark Gerstein
Peter Gething
Mahmoud Ghandi
Keyan Ghazi-Zahedi
Dario Ghersi
Geoffrey Ghose
Gourisankar Ghosh
Kingshuk Ghosh
Sean Gibbons
Stan Gielen
Carsten Giessing
Rona Giffard
Luis Gilarranz
Nick Gilbert
David Gilbert
Giorgio Gilestro
Jesse Gillis
Matthieu Gilson
Krzysztof Ginalski
Lisa Giocomo
Anthony Gitter
Michele Giugliano
Chad Giusti
Julijana Gjorgjieva
Stefan Glasauer
Kathryn Glass
Tilmann Glimm
Sebastian Gluth
Adam Godzik
Pranay Goel
Joshua Goldberg
Ido Golding
Byron Goldstein
Tim Gollisch
Leonardo Gollo
David Golomb
M. Gabriela Gomes
Shawn Gomez
Alexander Goncearenco
Joaquin Goni
Aitor Gonzalez
Marta Gonzalez
Didier Gonze
Benjamin Good
Kenneth Goodman
Anya Goodman
Manoj Gopalkrishnan
Uri Gophna
Raluca Gordan
Goren Gordon
Jeff Gore
Alemayehu Gorfe
Sara Gosline
Graeme Gossel
Raphael Gottardo
Mark Goulian
John Goutsias
Chris Govern

Sidhartha Goyal
Igor Goychuk
Frauke Gräter
Michael Grabe
Manfred Grabherr
Agnieszka Grabska-Barwinska
Daniel Graham
Trevor Graham
Eleonora Grandi
Pascal Grange
Barry Grant
Michael Graupner
Patti Gravitt
Frederik Graw
Richard Gray
Jeffrey Gray
Jack Gray
Warren Grayson
C. Shawn Green
David Greenberg
Sharon Greenblum
Bryan Greenhouse
C. Greenman
Joseph Greenstein
Nicolas Gregoire
Stephan Grein
Martin Greschner
Patrick Griffin
Thomas Griffiths
Carlos Grijalva
Kalanit Grill-Spector
Ramon Grima
Andrew Grimson
Nick Grishin
Gerrit Groenhof
James Grogan
M. Michael Gromiha
Alexander Grosberg
Allison Groseth
Ivo Grosse
Moritz Grosse-Wentrup
Alan Grossfield
Christian Grove
Helmut Grubmüller
Kristina Gruden
Sonja Gruen
Dmytro Grytskyy
Jenny Gu
Victor Guallar
Yuanfang Guan
Jeremie Guedj
Kristin Guertin
Emmanuel Guigon
William Guilford
James Gumbart
Rudiyanto Gunawan
Emre Guney
Ramneek Gupta
Todd Gureckis
Hugo Gutierrez de Teran
Michael Gutmann
Harendra Guturu
Oliver Hädicke
Jérôme Hénin
Felix Höfling
Katrin Hübner
Sauli Haataja
Suzana Hadjur
Ralf Haefner
Hiroshi Haeno
Jan Haerter
Tzachi Hagai
Daisuke Hagiwara
Stephanie Hagstrom
Matthew Hahn
Florian Hahne
Vincent Hakim
Turkan Haliloglu
Ian Hall
Timothy Hallett
Michael Hallett
Geir Halnes
Thomas Hamelryck
Daniel Hammer
Stavros Hamodrakas
Buhm Han
Andreas Handel
David Hansel
Dan Hansen
William Harcombe
Ian Harding
Marie Hardwick
Todd Hare
Simon Harris
Paul Harrison
Jennifer Harrow
HC Harsha
G Hart
Gal Haspel
Soha Hassoun
Alan Hastings
Stefan Haufe
James Havranek
Michael Hawrylycz
Satoru Hayasaka
Shigehiko Hayashi
Eric Hayden
John-Dylan Haynes
Steven Hayward
Vincent Hayward

William Hazelton
DaiHai He
David Heckmann
Tyson Hedrick
Dieter Heermann
Ines Heiland
Christine Heitsch
Richard Heitz
Brian Helmke
Volkhard Helms
Guillaume Hennequin
Matthias Hennig
Bruce Henry
Christopher Henry
Niel Hens
Burkhard Hense
Scott Hensley
Michael Henson
Jean-Karim Heriche
Rigoberto Hernandez
Esteban Hernandez Vargas
Miguel Herrero
Markus Herrgard
Andreas Herrmann
Eva Herrmann
Pascal Hersen
Uri Hershberg
Hanspeter Herzel
Peter Heutink
Stephen Hewitt
Matthew Hibbs
Kevin Hicks
Christian Hilbe
Mark Hildebrand
Sean Hill
Alison Hill
Grant Hill-Cawthorne
Ronald Hills
Jane Hillston
Konrad Hinsen
Tomoo Hirano
Lynette Hirschman
Martin Hjortsø
William Hlavacek
Vanthai Hoang
Birte Höcker
Toby Hocking
Ina Hoeschele
Michael Hoffman
Daniel Hoffmann
Sabrina Hoffmann
Kay Hofman
John Hogenesch
Arun Holden
Benjamin Holder
Guillaume Hollard
David Holloway
Petter Holme
Susan Holmes
Ian Holmes
Jeffrey Holmes
Hermann-Georg Holzhütter
Christian Hong
Barry Honig
Stefan Hoops
Thomas Hopf
Timothy Horiuchi
Jeremy Horst
Tingjun Hou
Conor Houghton
Thomas House
Eugene Houseman
Elizabeth Housworth
Martin Howard
Jonathon Howard
Clare Howarth
Brendan Howlin
Li Hsu
Jialu Hu
Xiao Hu
Zhenjun Hu
Gangqing Hu
Xuhui Huang
Niu Huang
Jeffrey Huang
Kerwyn Huang
Jing Huang
Jochen Hub
Greg Huber
Heinrich Huber
Matthew Hudson
Corey Hudson
Ramon Huerta
Sean Humbert
Gerhard Hummer
Mark Humphries
C. Antony Hunt
Daniel Huson
Cendri Hutcherson
Alexander Huth
Gyorgy Hutvagner
Quentin Huys
Eun Jung Hwang
Fahmeed Hyder
Changbong Hyeon
Timon Idema
Ales Iglic
Oleg Igoshin
Nevena Ilieva-Litova
Chris Illingworth

Princess Imoukhuede
Kiao Inthavong
Ed Ionides
Herve Isambert
Rezarta Islamaj Dogan
Yuval Itan
Viktor Ivanov
Junji Iwahara
Masoumeh Izadi
Dean Jackson
Robert Jacobs
Donald Jacobs
Anders Jacobsen
Matthew Jacobson
Johannes Jaeger
Frank Jaekel
Ajay Jain
Kavita Jain
Ciriyam Jayaprakash
Rick Jenison
Garrett Jenkinson
Lars Jensen
Oliver Jensen
Jan Jensen
Paul Jensen
Livnat Jerby Arnon
Robert Jernigan
Chris Jewell
Aaron Jex
Hongkai Ji
Shuiwang Ji
Wei Ji Ma
Yi Jiang
Hui Jiang
Bing-Hua Jiang
Yang Jiao
Alexandra Jilkine
Guangxu Jin
Dezhe Jin
Viktor Jirsa
Peter Johansen
Philip Johnson
Thomas Johnson
Henry Johnston
Wilsaan Joiner
Vladimir Jojic
Thibaut Jombart
Inge Jonassen
James Jones
Siddhartha Jonnalagadda
Rebecka Jornsten
Sanjay Joshi
Kresimir Josic
Daniel Jost
Peter Jung
Irwin Jungreis
Mordechai Juni
Ivan Junier
Lukas Käll
Jaap Kaandorp
Joe Kable
Lars Kaderali
Marcus Kaiser
Kostas Kalogeropoulos
Christel Kamp
Bjoern Kampa
Takahisa Kanekiyo
Shuli Kang
Ingmar Kanitscheider
Jitendra Kanodia
Eva Kanso
Rowland Kao
John Karanicolas
Jan Karbowski
George Karniadakis
Mikko Karttunen
Matthias Kaschube
Eugene Kashdan
Robert Kass
Peter Kasson
William Kath
Saul Kato
Michael Katze
Laura Kaufman
Kamran Kaveh
Kendrick Kay
Mitsunori Kayano
Hilal Kazan
Yiannis Kaznessis
Sunduz Keles
Douglas Kellogg
Daniel Kelly
J.A. Scott Kelso
Christopher Kempes
Eben Kenah
Terry Kenakin
Thomas Kepler
Andrew Kern
Rex Kerr
Can Kesmir
Sarah Kessans
Sinan Keten
Anmar Khadra
Ahmad Khalil
Fatemeh Khalili-Araghi
Mustafa Khammash
Himanshu Khandelia
Raya Khanin
Bhavin Khatri
George Khelashvili

Reza Khodarahmi
Roozbeh Kiani
Akinori Kidera
Andrzej Kierzek
Volker Kiessling
Daisuke Kihara
David Kikuchi
Zachary Kilpatrick
Yoo-Ah Kim
Jae Kyoung Kim
Philip Kim
Junhyong Kim
Taeyoon Kim
Yongsoo Kim
Sanguk Kim
Seyoung Kim
Marek Kimmel
Oliver King
Aaron King
Carl Kingsford
Tamara Kinzer-Ursem
Mark Kirkpatrick
Denise Kirschner
Istvan Kiss
Steffen Klamt
Per Johan Klasse
Jeffery Klauda
Max Kleiman-Weiner
Teri Klein
Ulrich Kleinekathoefer
Jurgen Kleine-Vehn
David Kleinfeld
Judith Klein-Seetharaman
Steven Kleinstein
Dmitri Klimov
David Klinke
Don Klinkenberg
Edda Klipp
Peter Kloeden
Yuval Kluger
Stefan Klumpp
Jonas Knape
David Knapp
Jefferson Knight
Dan Knights
David Knill
Andreas Knoblauch
Jaroslav Koča
Dmitry Kobak
Ina Koch
Peter Kochunov
Katia Koelle
Heinz Koeppl
Oliver Kohlbacher
Markus Kollmann
Rachel Kolodny
Anatoly Kolomeisky
Natalia Komarova
Michal Komorowski
Genevieve Konopka
Anna Konstor
Bette Korber
Tamás Korcsmáros
Alon Korngreen
Kirill Korolev
Sergei Kosakovsky Pond
Katya Kosheleva
Mickey Kosloff
Andrej Kosmrlj
Michael Kosorok
George Kostopoulos
Alexei Koulakov
Markos Koutmos
Roger Kouyos
Gregor Kovacic
Mehmet Koyuturk
Dima Kozakov
James Kozloski
Ian Krajbich
Mark Kramer
Bernhard Kramer
Natalio Krasnogor
Andreas Kremling
Robert Kretsinger
Elmar Krieger
Arjun Krishnan
Vessela Kristensen
Joachim Krug
Karsten Kruse
Sergey Kryazhimskiy
John Kubie
Roman Kuc
Andrzej Kudlicki
Arvind Kumar
Anshul Kundaje
Ambarish Kunwar
Chih-Horng Kuo
Daisuke Kurabayashi
Lukasz Kurgan
Shinya Kuroda
Edo Kussell
Rafal Kustra
Jason Kutch
Vladimir Kuznetsov
Taejoon Kwon
Inchan Kwon
David La
Giancarlo La Camera
Vincent Lacroix
Alain Laederach

Fereshteh Lagzi
Nicola Lai
Sirish Lakkaraju
Renaud Lambiotte
Jan Lammerding
Frank Lammert
Meytal Landau
Dirk Landgraf
Gary Landreth
Christian Landry
Samuel Landry
Michael Landy
Jenna Lang
Christopher Langmead
Daniel Larson
Michael Lassig
Peter Latham
Kenneth Latimer
Vito Latora
Andy Lau
Ken Lau
Reinhard C. Laubenbacher
Douglas Lauffenburger
Tim Laumann
Gilles Laurent
Michel Laurin
Christophe Lavelle
Richard Lavery
Jeffrey Lawrence
Michael Lawrence
Christopher Lawrence
Daniel Laydon
Themis Lazaridis
Matthew Lazzara
Nicolas Le Novère
Arthur Leblois
Paola Lecca
Gwenael Leday
Shane Lee
Dong-Yup Lee
Michael Lee
Jaejin Lee
Hyunju Lee
Sangwan Lee
Insuk Lee
Kyongbum Lee
Stefan Legewie
Joseph Lehar
Laurent Lehmann
Ming Lei
Hong Lei
Christian Leibold
Bertrand Lemasson
Louis Lemieux
Marc Lenburg
Máté Lengyel
Martin Lercher
Nicholas Lesica
Henry Leung
Erez Levanon
Gabriel Leventhal
Anna Levina
Michael Levitt
Sivan Leviyang
Emmanuel Levy
Yaakov Levy
Daniel Lew
Nathan Lewis
Bryan Lewis
Paul Lewis
Fran Lewitter
Klaus Ley
Lei Li
Hongzhe Li
Cong Li
Tong Li
Chunhe Li
Pan Li
Wei Li
Peng Li
Yue Li
Wei Li
Wenyuan Li
Jingjing Li
Li Li
Nan Li
Xiaoyu Liang
Han Liang
Jie Liang
Ran Libeskind-Hadas
Jonathan Licht
Oliver Lichtarge
Bomyi Lim
Jiaojiao Lin
Kevin Lin
Martin Linden
Stinus Lindgreen
Kresten Lindorff-Larsen
Michal Linial
Sébastien Lion
Tomasz Lipniacki
Veronique Lisi
Ashok Litwin-Kumar
Yunlong Liu
Tao Liu
Robert Liu
Song Liu
Dennis Livesay
Joseph Lizier
James Locke

Gerald Loeb
Benjamin Logsdon
Kirk Lohmueller
Nir London
Mickey London
André Longtin
Lit-Hsin Loo
Liliana Losada
Yoram Louzoun
Michael Love
Simon Lovell
David Lovell
Claude Loverdo
Pedro Lowenstein
Long Lu
Benzhuo Lu
James Lu
Xinghua Lu
David Lubensky
Sharon Lubkin
Joseph Lucas
Fabio Luciani
David Lukatsky
Desmond Lun
Daniel Lundin
Gerton Lunter
Jingqin Luo
Zewei Luo
Andrei Lupas
Javier Luque
G.W. Gant Luxton
Jeremy Lynch
Shina Caroline Lynn Kamerlin
Grant Lythe
Katrina Lythgoe
Alexander Lyubartsev
Lan Ma
Viktor Müller
Feilim Mac Gabhann
David MacAlpine
Ben MacArthur
Christopher MacDermaid
Malcolm MacIver
Jakob Macke
Alexander MacKerell
Paul Macklin
Jason MacLean
Shev MacNamara
Jeffry Madura
Carsten Magnus
Radhakrishnan Mahadevan
L. Mahadevan
Narendra Maheshri
Tarek Mahfouz
Volker Mai
Thomas Mailund
Francois Major
Alpeshkumar Malde
Sairam Mallajosyula
Thérèse Malliavin
Prema Latha Mallipeddi
Brian Malone
Pascal Mamassian
Piero Manfredi
Matthias Mann
Ranjan Mannige
Lisa Manning
Poramate Manoonpong
Manu
Luca Maragliano
Costas Maranas
Daniel Marbach
Adam Marblestone
Paolo Marcatili
Edward Marcotte
Eve Marder
Adil Mardinoglu
Andrea Mari
Daniele Marinazzo
John Marioni
Paul Marjoram
Alan E Mark
Doug Markant
Scott Markel
Florian Markowetz
Phineus Markwick
Siewert Jan Marrink
Joseph Marsh
Javier Martin
Alfonso Martinez-Arias
Marc Marti-Renom
Yosef Maruvka
Dominik Marx
Manja Marz
Laura Masgrau
Sergei Maslov
Christopher Mason
Timothée Masquelier
Marcello Massimini
Jean-Baptiste Masson
Francesco Massucci
Naoki Masuda
William Mather
Tom Matheson
Alexander Mathis
Frederick Matsen
Noriyuki Matsuda
Maurizio Mattia
Vincent Mauro
Eric May

Alberto Mazzoni
Mark McAuley
James McCaw
Megan McClean
Josh McDermott
Daniel Mcdonald
James McFarland
Joel McGlothlin
Alison McGuigan
Joseph McGuire
Alice McHardy
David McIver
William McLaughlin
Robert McMeeking
David McMillen
James McNally
Duccio Medini
Jan Medlock
Michael Mehan
Ahmed Mehdi
Ramit Mehr
Feryal Mehraban Pour Behbahani
Deavn Mehrotra
Pankaj Mehta
Martin Meier-Schellersheim
Hans Meinhardt
Manuel Melo
Francisco Melo
Raoul-Martin Memmesheimer
Pedro Mendes
Yilin Meng
Filippo Menolascina
Vinod Menon
Hugo Merchant
Dan Mercola
Vicky Merhej
Roeland Merks
Stefano Merler
Samy Meroueh
Martha Merrow
Arnaud Messé
C. Jessica Metcalfe
Dirk Metzler
Ralf Metzler
Markus Meuwly
Austin Meyer
Michelle Meyer
Michael Meyer-Hermann
Ethan Meyers
Rafael Meza
Hongyu Miao
George Mias
Anushka Michailova
Magali Michaut
Cristian Micheletti
Tom Michoel
Daniel Mietchen
David Miguez
Alexey Mikaberidze
Hiroaki Miki
Tijana Milenkovic
Olgica Milenkovic
Joel Miller
Yifat Miller
Lee Miller
Kai Miller
John Milton
Leonid Mirny
Yuriy Mishchenko
Natasa Miskov-Zivanov
John Mitchell
Colleen Mitchell
Julie Mitchell
Sorin Mitran
Satoru Miyano
Osamu Miyashita
Jeff Moehlis
Mohammad Mofrad
Alex Mogilner
James Moir
Jaap Molenaar
Sven Moller-Tank
Remi Monasson
Gianluigi Mongillo
Nick Monk
Ernest Montbrio
Enrique Monte
Stephen Montgomery
Luca Monticelli
M. Moody
Paul Moore
Thierry Mora
Rosalyn Moran
Massimo Morbidelli
Ruben Moreno-Bote
Kari Morgan
Martin Morgan
Dimitrios Morikis
Alexandre Morozov
Richard Morris
Quaid Morris
Richard Morton
Olaf Mosbach-Schulz
Roberto Mosca
Alan Moses
Mathieu Moslonka-Lefebvre
Sara Mostafavi
Rafal Mostowy
Fumio Motegi
Ahmed Moustafa

Andrés Moya
Aurelio Moya Garcia
Florian Mueller
Christian Mueller
Stephen Muench
Andrew Mugler
Sayan Mukherjee
Subhadeep Mukhopadhyay
Nicola Mulder
Sarah Muldoon
Adrian Mulholland
Christopher Mundt
Brian Munsky
Ivan Mura
Cyrill Muratov
Robert Murphy
Chris Murray
Philip Murray
Richard Murray
John Murray
Ben Murrell
Arvind Murugan
Ferdinando Mussa-Ivaldi
Ville Mustonen
Vivek Mutalik
Chris Myers
Chad Myers
Christopher Myers
Jean-Pierre Nadal
Zoltan Nadasdy
Elena Nadezhdina
Farzan Nadim
Suhita Nadkarni
Robert Nadon
Niranjan Nagarajan
Valentin Nagerl
Deepak Nagrath
John Nagy
Mate Nagy
Rafael Najmanovich
Suguru Nakagawa
Luay Nakhleh
Aurélien Naldi
Jin-Wu Nam
Kwangho Nam
Gautham Namasivayam
Atsushi Nambu
Olivier Namy
Giri Narasimhan
Rishikesh Narayanan
Jatin Narula
Thomas Naselaris
Matthew Nassar
Matt Nassar
Francisco Navarro
Kianoush Nazarpour
Peter Neal
Chris Neale
Emre Neftci
Richard Neher
Ali Neishabouri
Israel Nelken
Celeste Nelson
Peter Nelson
Ilya Nemenman
Goran Nenadic
Irina Nesmelova
Theoden Netoff
Thomas Nevian
Jay Newby
Stuart Newman
Fengyun Ni
Colin Nichols
Jeffrey Nickerson
Dan Nicolae
Stamatios Nicolis
Qing Nie
Steven Niederer
Jens Nielsen
Morten Nielsen
Evgeni Nikolaev
Roland Nilsson
Lennart Nilsson
Alex Ninfa
Hafumi Nishi
Chuanxin Niu
Masha Niv
Yael Niv
Frank Noé
Taishin Nomura
Rachel Norman
Sergei Noskov
Kirill Nourski
Houtan Noushmehr
Régis Nouvian
Daichi Nozaki
Sergey Nuzhdin
Mike Oaksford
Anna Ochab-Marcinek
Alejandro Ochoa
Lauren O'Connell
Gisele Oda
John O'Doherty
Lauren O'Donnell
Amy Odum
Thomas Oertner
Peter Oeschlager
Robert D Ogilvie
Uwe Ohler
Marie Öhman

Masato Okada
Eckehard Olbrich
Timothy O'Leary
Aude Oliva
Bruno Olshausen
Jose Onuchic
Thom Oostendorp
Hans Op de Beeck
Lulla Opatowski
Lance Optican
Jean-Jacques Orban de Xivry
Jill O'Reilly
Paul O'Reilly
Christine Orengo
Patricio Orio
Amy Orsborn
Pedro Ortega
Remus Osan
James Osborne
Anne Osbourn
Anne-Marie Oswald
Hans Othmer
Sarah Otto
Francis Ouellette
Thomas Ouldridge
Zhengqing Ouyang
Markus Owen
Dylan Owen
Diego Oyarzún
Ertugrul Ozbudak
Banu Ozkan
Elif Ozkirimli Olmez
Alberto Paccanaro
Roland Pache
Jorge Pacheco
Emanuele Paci
Christopher Pack
Andrea Pagnani
Debnath Pal
Sean Palecek
Günther Palm
Lucy Palmer
Stephanie Palmer
Arthur Palmer
Stefano Palminteri
Anna Panchenko
Liam Paninski
Jasmina Panovska-Griffiths
Tanate Panrat
Stefano Panzeri
Daniela Paolotti
Panagiotis Papasaikas
Jason Papin
Balázs Papp
Francesco Pappalardo
Leonardo Pardo
Néstor Parga
Laxmi Parida
Memming Park
Juyong Park
Christopher Park
Leopold Parts
Mercedes Pascual
Annalisa Pastore
Kiran Patil
Kaustubh Patil
Etienne Patin
Julie Pavlin
Gordana Pavlovic-Lazetic
Klaus Pawelzik
Florencio Pazos
Jean Peccoud
Morten Pedersen
Dana Pe'er
Shayn Peirce
Catherine Pellat-Deceunynck
Joelle Pelletier
Jian Peng
William Penny
John Pepper
Matjaz Perc
Jose Pereira-Leal
Alan Perelson
Alberto Perez
Alex Perkins
Sarah Perkins
Andrea Perna
Remo Perozzo
Robert Peterka
Megan Peters
Rasmus Petersen
Carsten Peterson
Karin Petrini
Giovanni Pezzulo
Jim Pfaendtner
Michael Pfeiffer
Benjamin Pfeuty
Jean-Pascal Pfister
Andrew Philippides
Francesco Piazza
Simone Pigolotti
Jonathan Pillow
Sergei Pilyugin
Flávio Pinheiro
Dimitris Pinotsis
Paulo Pinto
Davide Piovesan
Andrei Pisliakov

Xaq Pitkow
Francisco Planes
Mike Plank
Dietmar Plenz
Sylvia Plevritis
Maksim Plikus
Joshua Plotkin
Michael Poidinger
Panayiota Poirazi
Daniel Polani
Rafael Polania
Piero Poletti
Philip Polgreen
Régis Pomès
Adrian Ponce-Alvarez
Mikhail Ponomarenko
Beatriz Pontes
Art Poon
Tatiana Popova
Maurizio Porfiri
Francesc Posas
Juan Poyatos
Christian Pozzorini
Nataša Pržulj
Robert Preissner
Drazen Prelec
Tony Prescott
Steven Prescott
Steve Presse
Nathan Price
Astrid Prinz
U. Deva Priyakumar
William Probert
Sotiris Prokopiou
Vasilis Promponas
Nicholas Provart
Davide Provasi
Astero Provata
Paolo Provero
Przemyslaw Prusinkiewicz
Teresa Przytycka
Jingzhi Pu
Jose Puglisi
Alfredo Pulvirenti
Marco Punta
Jeremy Purvis
Feng Qi
Zhen Qi
Yuan Qi
Wenfeng Qian
Jiang Qian
Hong Qian
Yan Qin
Zhaohui Qin
Zhilin Qu
Changqin Quan
Gunnar Rätsch
Mikhail Rabinovich
Joshua Rabinowitz
Julien Racle
Todd Rae
Gregor Rainer
Arjun Raj
Sohini Ramachandran
Ravishankar Ramachandran
Kumaran Ramamurthi
E. Prabhu Raman
Stephen Ramsey
Padmini Rangamani
Rajiv Ranganathan
Christopher Rao
Nalam Rao
Benjamin Raphael
Wouter-Jan Rappel
Michael Rappolt
Maria Anna Rapsomaniki
Matthias Rarey
Garvesh Raskutti
David Rasmussen
Muruhan Rathinam
Oliver Ratmann
Thomas Rattei
Magnus Rattray
Erzsebet Ravasz Regan
Barak Raveh
J. Christian Ray
Soumya Raychaudhuri
Simon Rayner
Montague Read
Cyril Reboul
Pedro Reche
Mario Recker
Michael C. Reed
Jennifer Reed
Roland Regoes
Alexandra Reichenbach
Mark Reimers
Pieter Rein ten Wolde
Robert Reiner
Cynthia Reinhart-King
Johannes Reisert
Katarzyna Rejniak
Michiel Remme
Michael Rendl
Olivier Restif
Nathalie Reuter
Eduard Reznik
Seung Rhee
Nick Rhind
Ruy Ribeiro

Federico Ricci Tersenghi
Magnus Richardson
Stephen Richmond
Nicole Riddle
Andrea Riebler
Maximilian Riesenhuber
Guillem Rigaill
Michel Rigoulet
Steven Riley
Claire Rind
John Rinzel
Karsten Rippe
Herre Jelger Risselada
Marylyn Ritchie
Petra Ritter
Marcelo Rivas-Astroza
Vincent Robert
James Roberts
David Robertson
Richard Robins
Peter Robinson
Simon Robson
Walter Rocchia
Eduardo Rocha
Luis Rocha
Blanca Rodriguez
Isabel Rodriguez Barraquer
Pieter Roelfsema
Henk Roelink
Jose Rojas
Sandro Romani
Lilia Romdhane
Ethan Romero-Severson
Yannick Rondelez
Libin Rong
Angelo Rosa
Gabriel Rosser
Saharon Rosset
Simona Rossi
Igor Rouzine
Alex Roxin
Sushmita Roy
Christopher Rozell
Pau Rué
Leonid Rubchinsky
Dan Rubenstein
Joshua Rubin
Jonathan Rubin
Mikail Rubinov
Amir Rubinstein
Michael Rudd
Claus Rueffler
Matthew Ruffalo
Sandra Rugonyi
Peter Ruoff
Lasse Ruokolainen
Eytan Ruppin
Robert Russell
Michael Rust
Gustaf Rydell
Andrey Rzhetsky
Laura Sacerdote
Frank Sachse
Julio Saez-Rodriguez
Dov Sagi
Maneesh Sahani
Debashis Sahoo
Surinder S. Sahota
Naruya Saitou
Lisa Saksida
Kourosh Salehi-Ashtiani
Howard Salis
Alexandre Salvador
Julia Salzman
C. Daniel Salzman
Ines Samengo
Ilan Samish
Maria Samsonova
Karissa Sanbonmatsu
Roberto Sanchez
Conner Sandefur
Guido Sanguinetti
Michel Sanner
Vijayalakshmi Santhakumar
Francisco Santos
Mauro Santos
Vinay Satish Kumar
Uwe Sauer
Herbert Sauro
Olivier Saut
Thomas Sauter
Jeff Saven
Christina Savin
Nathaniel Sawtell
Akansha Saxena
Samuel Scarpino
Lars Schäfer
Jörg Schaber
William Schafer
Jeffrey Schall
Christoph Schaniel
Konrad Scheffler
Michael Schellenberger Costa
Birgit Schiøtt
Steven Schiff
Joshua Schiffer
Marcel Schilling
Mark Schira
Alexander Schliep
Patrick Schloss

Christoph Schmal
Sebastian Schmeier
Michael Schmuker
Ralf Schneggenburger
Birgit Schoeberl
Alexander Schoenhuth
Paul Schrater
Susanne Schreiber
Friederike Schueuer
Marcel Schulz
David Schulz
Peter Schuster
David Schwab
James Schwaber
David Schwartz
Daniel Schwartz
Russell Schwartz
Pamela Schwartzberg
Roland Schwarz
Nicolas Schweighofer
Michael Schwemmer
Joost Schymkowitz
Marco Scianna
Michelle Scott
Jacob Scott
David Searls
Timothy Secomb
Daniel Seeliger
Gunnar Seemann
Emre Sefer
Rebecca Segal
Nicola Segata
Ayellet Segrè
Terrence Sejnowski
Inbal Sela
Luc Selen
Guy Sella
Shamik Sen
Hanoch Senderowitz
Irene Sendina-Nadal
Alessandro Senes
Patrick Senet
Ryan Senger
Anirvan Sengupta
Walter Senn
Cathal Seoighe
Chaok Seok
James Sethna
Alessandro Sette
Manu Setty
Nikolaos Sgourakis
Shawn Shadden
Joshua Shaevitz
Patrick Shafto
Nigam Shah
Sohrab Shah
Premal Shah
Eugene Shakhnovich
Maoz Shamir
Shihab Shamma
Yibing Shan
Maryam Shanechi
Catherine Shang
Daryl Shanley
Tali Sharot
Gemma Sharp
Tatyana Sharpee
Thomas Sharpton
Jared Shaw
Eric Shea-Brown
Mark Shein
Jana Shen
Xiling Shen
Yanhong Shi
Xinghua Shi
Lei Shi
Darryl Shibata
Tetsuo Shibuya
Hideaki Shimazaki
Tom Shimizu
Yishai Shimoni
Jay Shin
Wataru Shinoda
Hiroki Shirai
Abbas Shirinifard
George Shirreff
G.V. Shivashankar
Eli Shlizerman
Mark Shlomchik
Harel Shouval
Jianwei Shuai
Igor Shuryak
Heike Siebert
Eric Siggia
Wendy Silk
Jerson Silva
Pamela Silver
Patrick Simen
Peter Simmons
Scott Simon
Eero Simoncelli
Daniel Simons
Thomas Simonson
Julie Simpson
Suzanne Sindi
Abhyudai Singh
Karan Singh
Sheila Singh
Saurabh Sinha
Photini Sinnis

Fabian Sinz
Jacobo Sitt
Per Sebastian Skardal
Maja Skataric
Peter Skelsey
Frances Skinner
Brian Skinner
Jan Skotheim
Brian Skyrms
Jamie Sleigh
Boris Slepchenko
Donna Slonim
Timo Smieszek
Roger Smith
Eric P. Smith
Maurice Smith
Tim Smith
V. Anne Smith
Amber Smith
Marcus Smolka
Michael Smotherman
Gordon Smyth
Erik Snapp
Michael Sneddon
Berend Snel
Kim Sneppen
James Sneyd
Jacky Snoep
Claudio Soares
Eric Sobie
Joshua Socolar
Johannes Soeding
Juan Soler
Soroosh Solhjoo
Trygve Solstad
George Somero
David Somers
Charlotte Soneson
Jimin Song
Sen Song
Nikolaus Sonnenschein
Andrew Sornborger
Cinque Soto
Christoph Sotriffer
Andrea Sottoriva
Andrew Spakowitz
Rainer Spang
Lee Spector
Michael Spivey
Jiri Sponer
Glen Spraggon
David Sprinzak
Sudharsan Sridharan
Jagan Srinivasan
Mandyam Srinivasan
Ganesh Sriram
Ranjan Srivastava
Peter Stadler
Dominic Standage
Kenneth Stanley
Anna Stary-Weinzinger
Oliver Stegle
Gary Stein
Dov Stekel
Federico Stella
Jorg Stelling
Ulrich Stelzl
Martin Stemmler
Klaas Stephan
Dagmar Sternad
Ralf Steuer
Alasdair Steven
Ian Stevenson
Klaus Stiefel
Gautier Stoll
Lewi Stone
Barbara Stranger
Birgit Strodel
Roland Strong
Michael Stumpf
Andrew Su
Shankar Subramaniam
Gurol Suel
Piotr Suffczynski
Sergei Sukharev
Christopher Summerfield
David Sumpter
Sean Sun
Margaret Sunde
Vikram Sunkara
Susan Sunkin
Richard Superfine
Granger Sutton
Samir Suweis
Maciej Swat
Gyorgy Szabo
Ewa Szczurek
Ágnes Tóth-Petróczy
Joel Tabak
Shoji Takada
Daniel Takahashi
Nobuto Takeuchi
Kai Tan
Ming Tan
Mark Tanaka
Sorin Tanase Nicola
Sishi Tang
Chao Tang
Yinjie Tang
Haixu Tang

Bertrand Tanner
Mounir Tarek
Peter Tass
Ozlem Tastan Bishop
Nicholas Tatonetti
Diethard Tautz
Iman Tavassoly
Merryn Tawhai
Jordan Taylor
Ronald Taylor
William Taylor
Christopher Taylor
Tracy Teal
James Tee
Tobias Teichert
Vladimir Teif
Marcus Teixeira
Aurelien Tellier
David Terman
Glenn Tesler
Bas Teusink
Shivendra Tewari
Johannes Textor
Mukund Thattai
Lucas Theis
Fabian Theis
Evangelos Theodorou
Frédéric Theunissen
Ines Thiele
Jean-Philippe Thivierge
Peter Thomas
Paul Thomas
Dennis Thomas
Morgane Thomas-Chollier
Wesley Thompson
Kurt Thoroughman
Simon Thorpe
Ian Thorpe
Xiao-Jun Tian
Chaoguang Tian
Alessandro Ticchi
D. Peter Tieleman
Axel Tiessen
Cezar Tigaret
Michael Tildesley
Marc Timme
Leleu Timothee
Brian Tjaden
Robert Tjian
Gašper Tkačik
Kai Toellner
Andreas Tolias
Dean Tolla
Iuliana Toma-Dasu
Giovanna Tomaiuolo
Cristian Tomasetti
Dan Tompkins
Tina Toni
Giulio Tononi
Maya Topf
Stefano Toppo
Bruce Torbett
Adriano Tort
Yoshihiro Toya
Taro Toyoizumi
Zlatko Trajanoski
Mark Transtrum
Cole Trapnell
Michael Tress
Jochen Triesch
Edward Trifonov
Antoine Triller
Wilson Truccolo
Anya Tsalenko
Krasimira Tsaneva-Atanasova
Doris Tsao
Lev Tsimring
Misha Tsodyks
Zhidong Tu
Elio Tuci
Lisa Tucker-Kellogg
Greg Tucker-Kellogg
Mark Tuckerman
Tamir Tuller
Nurcan Tuncbag
Paul Tupper
Srini Turaga
John Turchi
Adrian Turjanski
Joel Turner
Brandon Turner
Ten Tusscher
Jack Tuszynski
John Tyson
Balazs Ujfalussy
Nachum Ulanovsky
Igor Ulitsky
Jakob Ulmschneider
Myco Umemura
David Umulis
Peter Man-Un Ung
Marius Usher
David Ussery
Nagarajan Vaidehi
Sandor Vajda
Ady Vaknin
Ilya Vakser
Faramarz Valafar
Edelmira Valero Ruiz
Francisco Valero-Cuevas

Taufik Valiante
Robert van Beers
Willem van Berkel
Petra Van Damme
Martijn van de Bunt
Hugo Van den Berg
Martijn van den Heuvel
David van der Spoel
Arjan van der Vaart
Stijn van Dongen
Fred van Eeuwijk
Celia Van Gelder
Leo van Iersel
Erik van Nimwegen
Arjen van Ooyen
Jaap van Pelt
Natal van Riel
Mark van Rossum
Benjamin VanderSluis
Fabio Vandin
Rufin VanRullen
Jeffrey Varner
Charles Vaske
Stefano Vassanelli
Ilpo Vattulainen
Timothy Vaughan
Dimitrios Vavylonis
Catia Vaz
Gianluigi Veglia
Michele Vendruscolo
Paul Verschure
Jean-Philippe Vert
Cordula Vesper
Allegra Via
Cecile Viboud
Raul Vicente
Jonathan D. Victor
Brani Vidakovic
Marc Vidal
Edward Vigmond
Jose Vilar
Bjarni Vilhjalmsson
Arunachalam Vinayagam
Martin Vinck
Yngvild Vindenes
Alessia Visconti
Saraswathi Vishveshwara
Ivo Vlaev
Yoram Vodovotz
Vincent Voelz
Christopher Voigt
Raphael Voituriez
Erik Volz
Tobias von der Haar
Christof Von der Malsburg
Max von Kleist
Gregory Voth
Bradley Voytek
Edward Vul
Emilia Vynnycky
Thomas Wachtler
Rebecca Wade
Alex Wade
Eric-Jan Wagenmakers
Dylan Wagner
Aleksandra Walczak
Jerome Waldispühl
Patrick Walker
Michael Wall
Jeffrey Wall
Chris Wallace
Jacco Wallinga
Dirk Walther
Gilles Wandeler
Brian Wandell
Xiao Wang
Junwen Wang
DeLiang Wang
Jing Wang
Xiaowo Wang
Tao Wang
Ting Wang
Hongyun Wang
Zhihui Wang
Peter Wang
Qi Wang
Kai Wang
Xiao-Jing Wang
Bo Wang
Jin Wang
Xujing Wang
Rui Wang
John Wann
Melissa Ward
Joanna Wares
Andrew Wargo
Tandy Warnow
Tsjerk Wassenaar
Helen Wearing
Robert Weatheritt
Glenn Webb
Barbara Webb
Caleb Webber
Gerald Weber
Michael Webster
Matti Weckström
Wolfram Weckwerth
Michael Wehr
Gilbert Weidinger
Martin Weigt

Aaron Weimann
Ariel Weinberger
Patrick Weinkam
Harel Weinstein
Matthew Weirauch
Dahlia Weiss
Joshua Welch
Edward Wenger
Uno Wennergren
Wolfgang Wenzel
Benjamin Werner
Joel Wertheim
Jevin West
Pål Westermark
Robert Wheeler
Richard Wheeler
Nicole Wheeler
Chris Whidden
Laura White
John White
Paul Whitford
Steven Whitten
David Whitworth
Michael Wibral
Jeff Wickens
Andreas Wieland
Sadie Wignall
Herman Wijnen
Steven Wiley
Brian Wilhelm
Claus Wilke
Loren Williams
Ross Williamson
Robert Wilson
Bridget Wilson
Daniel Wilson
Jonathan Winawer
Erik Winfree
Ned Wingreen
Rudolf Winklbauer
Roland Winkler
Peter Winn
Patrick Wintrode
Thomas Wischgoll
Menno Witter
Judith Wodke
Florentin Woergoetter
Jana Wolf
Charles Wolgemuth
Thilo Womelsdorf
Chung Wong
James Wood
Michael Woodhams
Kim Worley
Michael Worobey
Geraldine Wright
Yu-Wei Wu
Yinghao Wu
Zhijin Wu
Joseph Wu
Alex Chi Wu
Wei Wu
Xuebing Wu
Haishan Wu
Dongying Wu
Stefan Wuchty
Christoph Wuelfing
Joao Xavier
Yu Xia
Yi Xiao
Bei Xiao
Xiao Xiao
Lu Xie
Xiaohui Xie
L Xie
Jinchuan Xing
Ren Xu
Wenfang Xu
Jinbo Xu
Li Xu
Larry Yaeger
Eitan Yaffe
Nir Yakoby
Yoshihiro Yamanishi
Brian Yandell
Wei Yang
Xingzhou Yang
Wan Yang
Ence Yang
Yang Yang
Tal Yarkoni
Michael Yarus
Andrew Yates
Jian Ye
Fei Ye
Todd Yeates
Tau-Mu Yi
Ahmet Yildiz
Kai Ying
Kevin Yuk-Lap Yip
Shibu Yooseph
Darrin York
Lingchong You
Peter Young
Alistair Young
Nada Yousif
Rolf Ypma
Pengfei Yu
Haibo Yu
Haiyuan Yu

Kebing Yu
Guo-Cheng Yuan
Karina Yusim
Ulrich Zachariae
Bojan Zagrovic
Qasim Zaidi
Alexey Zaikin
Andrew Zalesky
Naif Zaman
Roya Zandi
Bas-Jan Zandt
Ronen Zangi
Sara Zanivan
Jeremiah Zartman
Maria Zavodszky
Guenther Zeck
Peter Zee
Jonathan Zelner
Friedemann Zenke
Chang-Guo Zhan
Xuegong Zhang
Sulin Zhang
Lei Zhang
Chi Zhang
Jianzhi Zhang
Jing Zhang
Yu Zhang
Xian Sheng Zhang
Henggui Zhang
Xiaoyan Zhang
Le Zhang
Kechen Zhang
Shihua Zhang
Yuping Zhang
Shunan Zhang
Haiqing Zhao
Ting Zhao
Lili Zhao
Fangqing Zhao
Yi Zhao
Jie Zheng
Heping Zheng
Wenjun Zheng
Yujun Zheng
Deyou Zheng
Sheng Zhong
Joseph Zhou
Huan-Xiang Zhou
Yaoqi Zhou
Xiaobo Zhou
Baiyu Zhou
Changsong Zhou
Fangqiang Zhu
Victor Zhurkin
Piotr Zielenkiewicz
Nadine Ziemert
Ralf Zimmer
Christophe Zimmer
Andrei Zinovyev
Noam Ziv
Michal Zochowski
Ali Zomorrodi
Jinfeng Zou
Jun Zou
Paolo Zunino
Mark Zwart
Igor Zwir
Joel Zylberberg
